# Osimertinib retreatment for patients with advanced EGFR-mutated non-small cell lung cancer

**DOI:** 10.1016/j.ctarc.2025.101026

**Published:** 2025-10-25

**Authors:** Kevin M. Levine, Natalie F. Uy, Ted A. Gooley, Jenna Voutsinas, Micah Tratt, Keith D. Eaton, Rafael Santana-Davila, Alice H. Berger, Christina S. Baik

**Affiliations:** a University of Washington, Seattle, WA, USA; b Fred Hutchinson Cancer Center, Seattle, WA, USA

**Keywords:** Osimertinib, Retreatment, Rechallenge, Targeted therapy, Lung cancer

## Abstract

Resistance to osimertinib, a third-generation EGFR tyrosine kinase inhibitor, remains a key challenge in the treatment of EGFR-mutated non-small cell lung cancer (NSCLC). Although retreatment with earlier-generation EGFR tyrosine kinase inhibitors has been studied, data on osimertinib rechallenge are limited. We conducted a single institution retrospective analysis of patients with EGFR-mutated NSCLC who were rechallenged with osimertinib following progression on prior osimertinib and interim systemic therapy, including chemotherapy. Seventeen patients met inclusion criteria, all with adenocarcinoma histology and either EGFR exon 19 deletions or L858R mutations. Median interval between osimertinib treatments was 10.5 months. Osimertinib retreatment resulted in a partial response in 18 % (3/17) and stable disease in 35 % of patients (6/17), for a disease control rate of 53 % (9/17). Median retreatment duration was 4.3 months, and median overall survival following retreatment initiation was 8.9 months. Among patients with central nervous system involvement, several experienced intracranial stability without additional radiation. Duration of initial osimertinib therapy did not strongly predict retreatment benefit. These findings suggest that osimertinib rechallenge may provide clinically meaningful disease control in a subset of patients, especially given its tolerability and oral formulation. Further research is needed to identify biomarkers of sensitivity and to optimize patient selection for osimertinib retreatment following interval chemotherapy.

## Introduction

Osimertinib is a third-generation epidermal growth factor receptor (EGFR) tyrosine kinase inhibitor (TKI) approved as front-line therapy for patients with advanced non-small cell lung cancer (NSCLC) whose tumors harbor activating *EGFR* mutations [1]. Osimertinib has demonstrated excellent and durable treatment response rates with a favorable side effect profile; however, nearly all patients eventually acquire resistance and develop disease progression. Treatment after front-line osimertinib for advanced EGFR-mutant NSCLC remains a clinical challenge. Combination chemotherapy is frequently used as subsequent-line therapy, and with recent data from the MARIPOSA-2 trial, clinicians can also consider the addition of amivantamab, a bispecific EGFR-MET antibody [2,3]. Although there are ongoing clinical trials in this space for 4th generation EGFR inhibitors and additional MET inhibitors, the rate of on-target osimertinib resistant mutations, such as *EGFR* C797S, and MET amplification together represent the minority of patients with osimertinib resistance [4]. In an analysis of the FLAURA trial, 10 % of patients were identified to have acquired *EGFR* resistance mutations and 16 % were identified to have MET amplifications on progression to osimertinib [4].

Treatment rechallenge after initial disease progression and interval systemic therapy has been studied with earlier generation EGFR TKIs [5–9]. As described in a recent meta-analysis by Michelon et al. [10], this is a reasonable strategy, with median overall survival being approximately 12 months following rechallenge with a 1st or 2nd EGFR TKI. However, in comparison, there are very few case reports on osimertinib retreatment [11], with the largest retrospective case series of 17 Asian patients showing a median overall survival of 9 months following osimertinib rechallenge [12]. The rationale for EGFR TKI retreatment is that interval chemotherapy may eradicate the clones responsible for EGFR TKI resistance [13]. Given the low rate of on-target *EGFR* resistance mutations to osimertinib, it is reasonable to consider that osimertinib rechallenge may have more success than rechallenge to earlier generation TKIs, where on-target *EGFR* mutations (T790M) are much more common [4]. Here, we describe outcomes of patients with EGFR-mutated NSCLC in a single institution who received osimertinib in the retreatment setting.

## Methods

Patients with confirmed EGFR-mutated NSCLC were retrospectively reviewed from a single institutional database from January 1, 2010-December 31, 2022. Primary objectives were to define response rate, duration of osimertinib rechallenge, and overall survival following start of osimertinib rechallenge. We identified patients who received osimertinib monotherapy in the retreatment setting after interval systemic therapy following initial disease progression while on osimertinib. Patients were included whether they initially received osimertinib as first or subsequent line of therapy. No patients received adjuvant osimertinib in this cohort. Patients who were rechallenged after holding osimertinib due to adverse effects without disease progression were excluded. Any *EGFR* mutation was eligible for inclusion in this study. Patient characteristics and tumor type were collected via chart review. Medical records were reviewed until either the patient was deceased or lost to follow up. Disease response to osimertinib rechallenge was determined as best response using RECIST 1.1 criteria on restaging scans. Duration of osimertinib rechallenge was defined as time from retreatment start to date of last dose, via clinical chart review. Overall survival was defined as time from retreatment start to either date of death, or if lost to follow up, patients were censored at date of last contact. Median overall survival and confidence intervals were calculated using the Kaplan-Meier method. Correlation was calculated using Pearson’s method. Statistical analysis was performed using R version 4.3.1.

## Results

All patients from an internal Fred Hutch database that were treated between January 1, 2010 and December 31, 2022 were included for consideration in this retrospective review. This included 2024 patients with lung adenocarcinoma and 449 with *EGFR* mutations. Of these, 209 patients had received osimertinib at least once in the designated time-frame and 17 patients met the above inclusion criteria for osimertinib rechallenge. Reasons for exclusion were the use of osimertinib in the adjuvant setting and discontinuation for adverse effects rather than progression. Two patients received osimertinib retreatment twice, and only the first retreatment was included in detailed analyses. Median age at time of osimertinib retreatment was 62 years (range 42–74). Patients were predominantly female (*n* = 11, 65 %), white (*n* = 10, 59 %) or Asian (*n* = 7, 41 %), and never smokers (*n* = 13, 76 %) (Table 1).

All patients had either an *EGFR* exon 19 deletion (*n* = 11, 65 %) or L858R mutation (*n* = 6, 35 %). Four patients received osimertinib as first line systemic therapy (24 %). Twelve patients (71 %) received a 1st or 2nd generation EGFR TKI (erlotinib *n* = 10, 59 %, afatinib *n* = 2, 12 %) prior to initial osimertinib, and of these, 8 (47 %) had an additional T790M mutation. Ten patients (59 %) had CNS metastases prior to osimertinib retreatment, of which 3 had leptomeningeal involvement. Eleven patients (65 %) had ECOG 0–1 at time of retreatment.

**Fig. 1.**
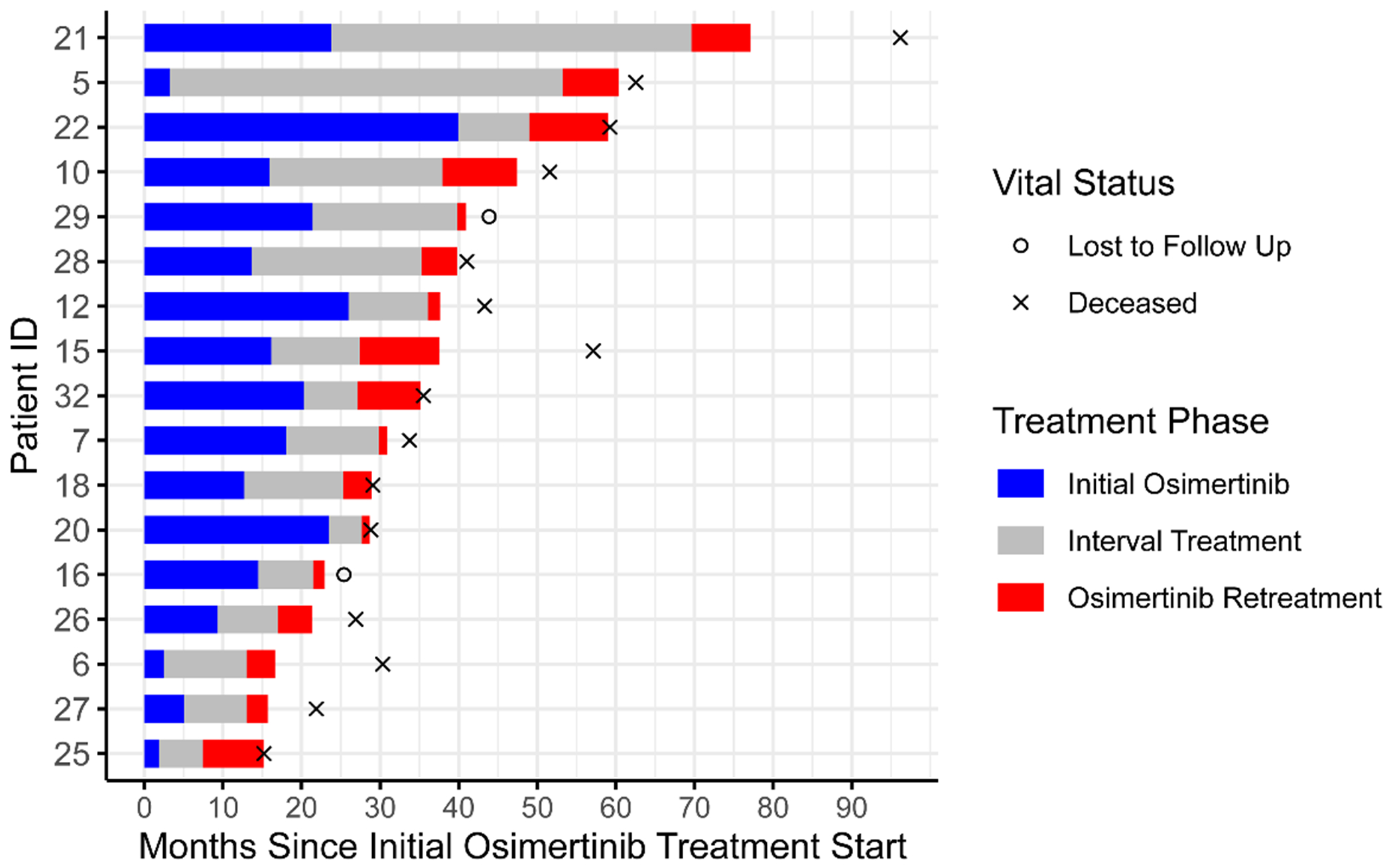
Swimmer plot of patient outcomes showing osimertinib treatment course.

The median interval time between initial osimertinib discontinuation and osimertinib rechallenge was 10.5 months (range 4.2–50 months). Patients had a median of 4 (range 2–5) total lines of systemic therapy prior to osimertinib rechallenge, including a median of 2 (range 1–3) lines of systemic therapy in the interval between initial and rechallenge treatment. All 17 patients (100 %) received interval chemotherapy. Most often, this was a combination of carboplatin and pemetrexed, but regimens also included single-agent pemetrexed, combination carboplatin and gemcitabine, paclitaxel, and docetaxel. In addition, 2 (12 %) received interval immune checkpoint inhibitor monotherapy, 3 (18 %) received interval antibody drug conjugates, and 8 (47 %) received interval palliative radiation or palliative surgery plus radiation (Fig. 1 and Table 1).

Median duration of initial osimertinib treatment was 15.9 months (range 1.9–39.9 months), and median treatment duration in the rechallenge setting was 4.3 months (range 1.0–10.2 months). Among 9 patients who achieved disease control in the retreatment setting, median retreatment duration of osimertinib was 7.5 months (range, 2.7–10.2 months). Eleven (65 %) patients received osimertinib retreatment for at least 3 months, and 7 (41 %) received osimertinib retreatment for at least 6 months. Median overall survival was 8.9 months (95 % confidence interval, 5.8–13.6 months) after osimertinib retreatment start date.

There was a weak correlation between the duration of first osimertinib treatment and duration of osimertinib retreatment (Pearson *r* = 0.10, *p* = 0.7), with initial duration longer, on average, than retreatment duration (median difference, 9.1 months, range −5.9 to 29.9 months). There was also only a weak correlation between interval treatment length and duration of osimertinib retreatment (Pearson *r* = 0.28, *p* = 0.28).

Median duration of osimertinib rechallenge for the 4 patients who received initial osimertinib as 1st line systemic therapy was 4.4 months (range 3.6–7.1 months), similar to the median duration for initial osimertinib in later line settings (3.6 months, range 1.0–10.2 months).

Among the 8 patients who received interval local therapy, including palliative radiation alone or palliative surgery with post-op radiation, the mediation duration of osimertinib rechallenge was 3.6 months (range 1.0–9.5 months), similar to the duration for the 9 patients without interval local therapy (4.6 months, range 1.1–10.2 months).

Fifteen patients had follow-up scans after re-initiation of osimertinib; 1 died before imaging was obtained due to disease progression and 1 did not get additional imaging due to patient preference. Among the entire cohort of 17 patients, 3 achieved partial response (PR) (18 %), and 6 patients had stable disease (SD) as the best response (35 %), for an overall disease control rate of 53 %. There was not a clear association between the likelihood of PR and *EGFR* mutation type (exon 19 deletion vs L858R), initial duration of osimertinib treatment, interval duration of treatment, total lines of systemic therapy, or duration of osimertinib rechallenge. Of note, obtaining a PR did not predict prolonged overall survival, as the median overall survival after osimertinib retreatment start date for these 3 patients was 8.9 months, identical to the cohort as a whole.

Among 10 patients who had CNS involvement, 9 had active disease at time of retreatment. Four patients received palliative CNS radiation treatment prior to osimertinib retreatment. Five patients received osimertinib retreatment only without additional radiation; on brain MRI imaging, 1 had progressive disease, 3 had stable or improved intracranial disease burden, and 1 did not have follow up brain imaging. Response categories were determined by the clinical radiologist impression on the first brain MRI in the retreatment setting, rather than standardized RECIST criteria, as RECIST reports were not available.

Osimertinib treatment was well tolerated, with the most common side effects being diarrhea (*n* = 5, 29 %) and rash (*n* = 2, 12 %). No patients had to dose reduce or discontinue osimertinib due to side effects.

**Fig. 2.**
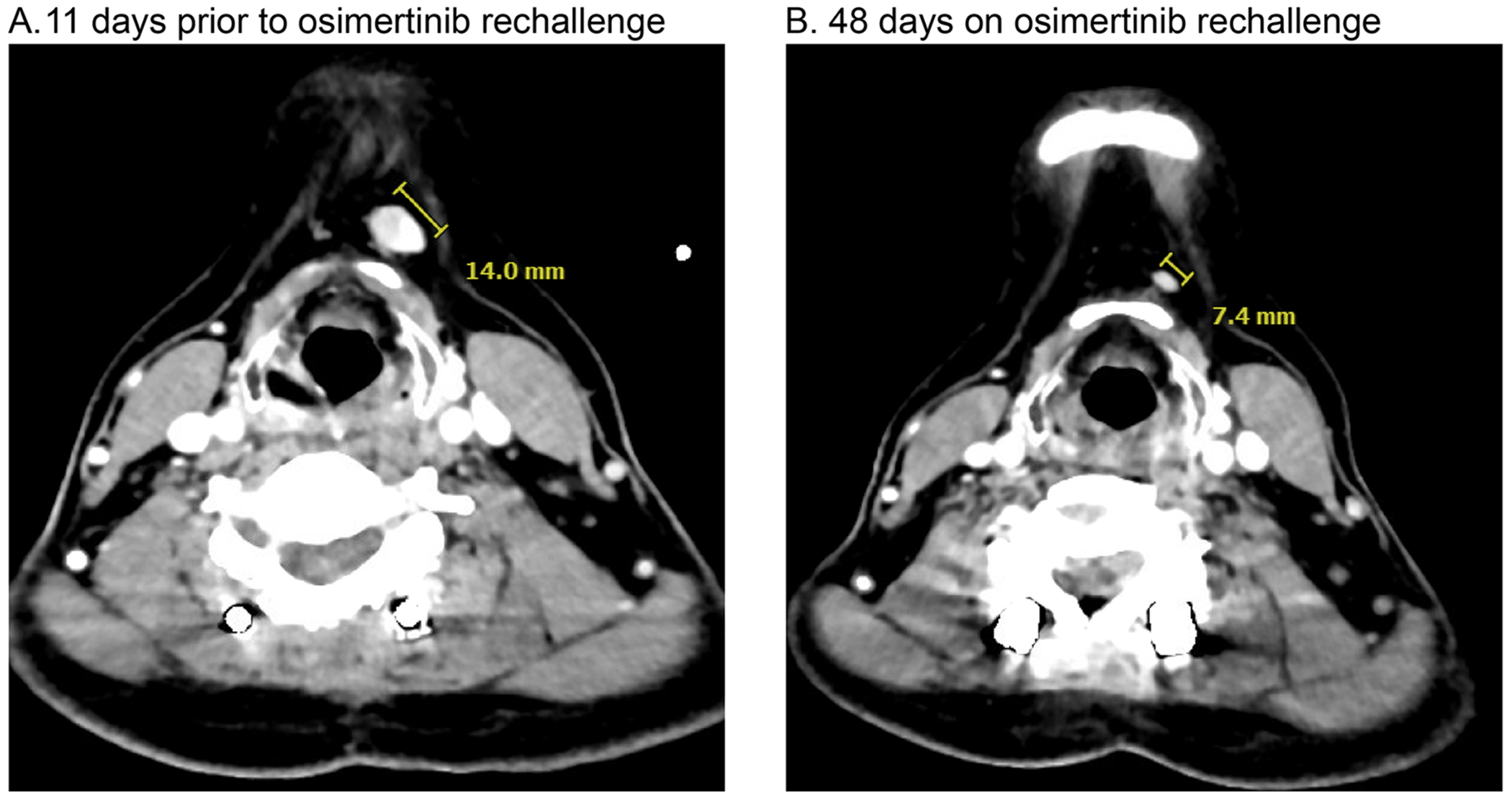
Radiographic response for patient 5 for osimertinib rechallenge. A) CT Neck w/ contrast image shows a 14 mm left level 1A submental lymph node 11 days prior to osimertinib rechallenge. B) CT Neck w/ contrast image shows that this lymph node has decreased in size to 7 mm 48 days following re-initiation of osimertinib.

An example of a patient with a remarkable response to osimertinib rechallenge is patient 5, who had an initial osimertinib treatment duration of only 3 months prior to radiologic progression (Fig. 1). Interval duration was 50 months, with treatments including chemotherapy, immunotherapy, surgery for pathologic fractures, and palliative radiation. On rechallenge, they had significant clinical improvement by 3 weeks, with notable decrease in cervical lymph node (Fig. 2) and tongue metastasis size, in addition to improved cough and fatigue. They remained on osimertinib in the rechallenge setting for 7 months until disease progression.

Three patients had genomic testing in the interval between initial osimertinib progression and rechallenge. Only one of these, patient 7, had an identified resistance mutation to osimertinib, *EGFR* L718Q. In the 12 month interval period, they were treated with chemotherapy, including carboplatin, gemcitabine, and paclitaxel. Of note, this patient had one of the shorter durations of osimertinib rechallenge at 1.1 months. They did not have subsequent genomic testing to assess if the L718Q mutation was still present at time of osimertinib rechallenge.

## Discussion

With recent data from the MARIPOSA-2 trial, amivantamab plus chemotherapy is now an available treatment option for patients with EGFR-mutated NSCLC after progression on osimertinib [2]. However, median progression-free survival in that trial was only 6.3 months by blinded independent central review [3]. Additional treatment options for this patient population include datopotamab deruxtecan, which received accelerated FDA approval in June 2025. This approval was based partly on data from the TROPION-Lung05 study, which found a similar median progression-free survival of 5.8 months for patients with *EGFR* mutations [14]. Therefore, there remains a significant clinical need for efficacious treatment options following progression on osimertinib. Here, we report our institution’s outcomes for osimertinib in the retreatment setting, specifically including 17 patients who had interval treatment with chemotherapy, with or without additional systemic or local therapies.

Some studies with earlier generation TKIs suggest that a longer TKI free interval may predict better outcomes with retreatment [15,16], although there is also data showing no correlation between first and second response duration[17]. In this study, we report a very weak correlation (*r* = 0.10) between osimertinib treatment durations, less than that seen in the only other study looking at osimertinib in the retreatment setting (*r*= 0.59) [12]. Of note, prolonged response to initial osimertinib treatment was not included as an eligibility criteria, but 16/17 patients had at least 3 months of treatment with an EGFR TKI prior to osimertinib rechallenge.

In terms of treatment response, the prior osimertinib rechallenge data from a single Japanese institution reported by Ichihara et al. found an ORR of 33 %, disease control rate (DCR) of 73 %, and a median overall survival of 9.0 months [12]. Our study found a lower ORR (18 %) and DCR (53 %), but a similar median overall survival of 8.9 months.

Chemotherapy kills rapidly dividing cells preferentially, so it is hypothesized that without the selective pressure of EGFR TKIs, EGFR pathway sensitivity can be restored upon disease progression via several mechanisms [5]. As an example, a patient’s tumor may have developed heterogenous resistance to osimertinib, with some cells acquiring an *EGFR* C797S mutation that prevents osimertinib binding, and some cells entering a more quiescent, or drug-tolerant persister state [18]. Cells with the C797S mutation would be hypothesized to grow quicker than the more quiescent persister cells without the mutation and therefore be more sensitive to chemotherapy [19]. At time of progression to chemotherapy, it may be those drug-tolerant persister cells without the *EGFR* C797S mutation that have survived and found an alternative mechanism to grow, of which some may have renewed sensitivity to EGFR inhibition. Based on preclinical studies of drug-tolerant persister cells, further research is warranted to better understand which patients are likely to benefit from EGFR TKI rechallenge [19,20]. In our study, only 3/17 patients had genomic testing in the interval between initial osimertinib treatment and rechallenge. For future studies with a higher proportion of available genomic data, it would be interesting to assess the utility of EGFR TKI rechallenge in the setting of on-target vs off-target resistance mechanisms.

The limitations of this study include the fact that it is a single-institution retrospective case series. By its nature, this study includes a small number of patients and lacks a standardized methodology for imaging follow up to determine response. There are multiple potential sources of bias for patient selection in terms of who may have been chosen to pursue osimertinib rechallenge. For example, our retrospective case series may be enriched for patients with CNS disease, since osimertinib is known to have good brain penetration and CNS disease may have been a barrier for entry into other ongoing clinical trials. Our dataset includes 6/17 patients with a documented ECOG performance status of 2 or greater and so should be interpreted carefully against clinical trials with more strict entry criteria. A further potential source of bias is that patients may have been less likely pursue osimertinib rechallenge if their initial treatment course of osimertinib had limited clinical benefit or a poor side effect profile. Overall, the results must be interpreted with an understanding that this is a retrospective case series and taken in the context of the prior case series of EGFR TKI rechallenge.

## Conclusions

Osimertinib retreatment is a potential option for patients with advanced EGFR-mutated NSCLC to obtain disease control following interval chemotherapy. In this study, we show that 3/17 patients had a partial response to osimertinib rechallenge, with disease control in 9/17 patients. Further research is needed to better select which patients will benefit from EGFR TKI re-exposure, but osimertinib’s relative tolerability, oral formulation, and potential for a clinically significant duration of response make it an attractive option for a subset of patients.

## Figures and Tables

**Table 1:** Patient characteristics.

Patient characteristics at time of osimertinib retreatment	*N* = 17
Median Age in years (range)	62 (42–74)
Sex	
Male	6 (35 %)
Female	11 (65 %)
Race	
White	10 (59 %)
Asian	7 (41 %)
Stage at Diagnosis	
I-III	2 (12 %)
IV	15 (88 %)
EGFR Mutation	
Del19	11 (65 %)
L858R	6 (35 %)
ECOG at Time of Retreatment	
0–1	11 (65 %)
>=2	6 (35 %)
Treatment Line of Initial Osimertinib	
1st Line Osimertinib	4 (24 %)
>= 2nd Line Osimertinib	13 (76 %)
Median Lines of Therapy Prior to Retreatment (range)	4 (2–5)
Median Interval of Lines of Systemic Therapy (range)	2 (1–3)
Median Duration Between Initial and Retreatment Osimertinib in Months (range)	10.5 (4.2–50)
Interval Systemic Therapy Regimens	
Chemotherapy	17 (100 %)
Immunotherapy	2 (12 %)
Antibody Drug Conjugate	3 (18 %)
Interval Local Therapy Received	8 (47 %)
Palliative Radiation Alone	6 (35 %)
Palliative Surgical Resection and Radiation	2 (12 %)

ECOG, Eastern Cooperative Oncology Group Performance Status; Del19, exon 19 deletion mutation; L858R, exon 21 L858R point mutation.

## Data Availability

Data will be made available under reasonable request to the corresponding author.
